# Synthesis and Investigation of the Hydration Degree of CA_2_ Phase Modified with Boron and Fluorine Compounds

**DOI:** 10.3390/ma17092030

**Published:** 2024-04-26

**Authors:** Michał Pyzalski, Karol Durczak, Agnieszka Sujak, Michał Juszczyk, Tomasz Brylewski, Mateusz Stasiak

**Affiliations:** 1 Faculty of Materials Science and Ceramics, AGH University of Krakow, Al. Mickiewicza 30, 30-059 Cracow, Poland; brylew@agh.edu.pl; 2 Department of Biosystems Engineering, Faculty of Environmental and Mechanical Engineering, Poznan University of Life Sciences, Wojska Polskiego 50, 60-627 Poznan, Poland; karol.durczak@up.poznan.pl (K.D.); agnieszka.sujak@up.poznan.pl (A.S.); 3 Faculty of Civil Engineering, Cracow University of Technology, 31-155 Cracow, Poland; michal.juszczyk@pk.edu.pl; 4 Institute of Agrophysics, Polish Academy of Sciences, Doświadczalna 4, 20-290 Lublin, Poland; m.stasiak@ipan.lublin.pl

**Keywords:** calcium aluminate, fluoride compound, mineralizing additives, boron compound, hydration degree, synthesis temperature

## Abstract

This study investigated the effect of fluoride and boron compound additives on the synthesis and hydration process of calcium aluminate (CA_2_). The analysis showed that the temperature of the full synthesis of CA_2_ without mineralizing additives was 1500 °C. However, the addition of fluorine and boron compounds at 1% and 3% significantly reduced the synthesis temperature to a range of 1100–1300 °C. The addition of fluoride compounds did not result in the formation of fluoride compounds from CaO and Al_2_O_3_, except for the calcium borate phase (Ca_3_(BO_3)2_) under certain conditions. In addition, the cellular parameters of the synthesized calcium aluminate phases were not affected by the use of these additives. Hydration studies showed that fluoride additives accelerate the hydration process, potentially improving mechanical properties, while boron additives slow down the reaction with water. These results highlight the relevance of fluoride and boron additives to the synthesis process and hydration kinetics of calcium aluminate, suggesting the need for further research to optimize their application in practice. TG studies confirmed the presence of convergence with respect to X-ray determinations made. SEM, EDS and elemental concentration maps confirmed the presence of a higher Al/Ca ratio in the samples and also showed the presence of hexagonal and regular hydration products.

## 1. Introduction

Aluminate cement is a rapidly setting, high-strength hydraulic material, consisting mainly of calcium aluminates. The main crystalline phases in aluminate cements include monocalcium aluminate CaO·Al_2_O_3_ (CA) and dicalcium aluminate CaO·2Al_2_O_3_ (CA_2_), as well as calcium ferrite and calcium aluminoferrite of the type 2CaO·Fe_2_O_3_ (C_2_F), 4CaO·2Al_2_O_3_·Fe_2_O_3_ (C_4_AF). Additionally, in smaller quantities, phases such as 12CaO·7Al_2_O_3_ (C_12_A_7_), 2CaO·SiO_2_ (C_2_S), or 2CaO·2Al_2_O_3_·SiO_2_ (C_2_AS) are present [1,2,3,4]. Literature review [5,6] indicates that the physicochemical characteristics of aluminate cements, such as strength and setting times, mainly depend on the content of the CA phase and its properties. The dicalcium aluminate phase typically exhibits lower reactivity with water compared to the CA phase. Increasing the content of the CA_2_ phase leads to improved refractoriness of the material and the final strength of concretes prepared with such cement [5].

These types of cement are among the high-performance binders characterized by a very rapid increase in initial strength. After only a dozen hours the strength for serviceability is reached. A major advantage of these cements is also their resistance to high temperatures and ability to be used in operations carried out in winter conditions due to their significant heat of hydration. They also show satisfactory durability in waters rich in various mineral salts. They are relatively resistant to weak acids, CO_2_-rich waters, and industrial effluents. Aluminous cements are used in the production of expansive cement, in the metallurgy, energy and chemical industries for various types of concrete and concrete masses operating at high temperatures (even above 1700 °C). In the mining industry, they are used for repair and construction work, injection and pit protection. The disadvantage of these cements is their poor resistance to alkaline solutions. Unfortunately, the production cost of aluminous types of cement is many times that of Portland cement [5].

The physicochemistry of aluminous cements is integrally linked to the CaO-Al_2_O_3_ system and requires detailed analysis. Work on this system has been the subject of numerous studies and the results obtained often show inconsistencies. It should be noted that this system is not yet fully understood and described. There are several calcium aluminates in this system including C_3_A, C_12_A_7_, CA, CA_2_ and CA_6_. C_12_A_7_ was previously referred to as C_5_A_3_, while CA_2_ as C_3_A_5_ [5].

Most researchers believe that C_3_A and CA_6_ melt incongruently, but opinions differ on the melting of the other aluminates. Nurse’s research has shown that calcium aluminates melt incongruently, while Kravchenko reports that C_12_A_7_ occurs in cement as a unstable variety, and that stability is reached upon heating. Other authors claim that calcium dodecaluminate has polymorphic varieties, the transition of which is observed in the temperature range 1185–1305 °C. Some researchers believe that CA melts incongruently, forming a liquid similar in composition to the theoretical chemical composition of CaO-Al_2_O_3_. Others, however, suggest that calcium monoaluminate melts congruently, forming a liquid with a composition corresponding to the theoretical chemical composition of calcium aluminate [5,6]. The most important physicochemical properties of the phases of the CaO-Al_2_O_3_ system are shown in Table 1 and Table 2.

The properties of aluminate cements depend on their chemical and phase composition and the individual properties of each phase. Understanding the processes occurring between water and the dicalcium aluminate compound CA_2_ is crucial for comprehending the essential physicochemical properties of refractory concrete. Comparative studies regarding the speed and dynamics of the hydration processes of CA_2_ compound preparations, with the addition of temperature synthesis-reducing mineralizers, are particularly interesting and not yet fully explored [7,8]. The addition of various types of mineralizers (additives) typically alters the characteristic temperatures of mineral formation or melting during their thermal processing. These additives may lead to the formation of solid solutions, defecting the structure, the appearance of polymorphic varieties, or the creation of new phases, which consequently affect the changes in the physicochemical properties of the newly formed compounds [9]. Understanding the structure of minerals, especially those contained in aluminate clinkers with additives such as boron and fluorine compounds, is significant from the perspective of cement chemistry, mainly due to the reduction in the temperature of their formation, which will also influence sustainable development phenomena [10].

The addition of boron compounds to calcium aluminates, such as C_12_A_7_ and CA, results in the decomposition of these phases and the formation of new minerals [11,12]. In the case of C_12_A_7_ samples, the formation of calcium borate Ca_3_(BO_3_)_2_ and the CA phase adjacent to the parent phase is observed. The content of Ca_3_(BO_3_)_2_ and CA phases varies with the amount of added boron. Therefore, it can be inferred that the addition of boron to the C_12_A_7_ phase results in its decomposition into CA and unbound CaO, which then reacts with B_2_O_3_ to form Ca_3_(BO_3_)_2_ [13]. The course of the reaction likely proceeds as follows:

3(12CaO·7Al_2_O_3_) + 5(B_2_O_3_) → 5[Ca_3_(BO_3_)_2_] + 21(CaO·Al_2_O_3_) (1)

In the case of doping monocalcium aluminate with boron compounds, the coexistence of the CA_2_ phase and Ca_3_(BO_3_)_2_ was observed. The probable course of the reaction is as follows:

6(CaO·Al_2_O_3_) + B_2_O_3_ → Ca_3_(BO_3_)_2_ + 3(CaO·2Al_2_O_3_) (2)

Analysis of the binary systems CaO-B_2_O_3_ and Al_2_O_3_-B_2_O_3_ in the temperature range from 1100 °C to 1500 °C has shown that with the addition of boron in the range of 1% to 3% (by weight), compounds such as 3CaO·B_2_O_3_+CaO and liquid phase can coexist. Similarly, in the Al_2_O_3_-B_2_O_3_ system, under the same boron content and temperature range, compounds like 9Al_2_O_3_·2B_2_O_3_ and liquid phase are observed [13,14].

So far, there are no relevant scientific publications regarding the doping of calcium aluminate with fluorine compounds at levels from 1% to 3% (by weight), synthesized in the temperature range from 1100 °C to 1500 °C. There is only literature data concerning the relationship between CaF_2_ and AlF_3_ compounds, which has limited significance for the present study [13,15].

This research paper discusses the influence of mineralizers (additives) in the form of boron or fluorine compounds on the synthesis temperature of the calcium aluminate phase. The study aims to present, analyze, and discuss preliminary experimental investigations concerning the hydraulic activity of calcium aluminate compounds modified with selected boron or fluorine compounds. The obtained results serve as a starting point for further research on the crystal chemistry of calcium aluminate compounds modified with boron oxide and fluorine oxide, as well as their impact on hydraulic activity and refractoriness.

## 2. Materials and Methods

### 2.1. Samples Preparation

It was assumed that calcium aluminate would be obtained from chemically pure compounds (CP) CaCO_3_ and Al_2_O_3_ (POCH, Poland S.A., Gliwice, Poland). Then, the calcination losses of the used compounds were determined, which amounted to −43.57% for CaCO_3_ and −3.29% for Al_2_O_3_, respectively. From the weighted and combined compounds, taking into account their calcination losses, a raw material set was prepared. This set was then subjected to homogenization in a rotary mixer prepared for this purpose for a period of 20 h [16,17].

The next stage of homogenization was continued in spherical agate mills with the addition of distilled water (to the consistency of a slurry). After the homogenization process was completed and the mixture was dried, the obtained raw material set was subjected to preliminary heat treatment at 100 °C for 30 min [3,18].

It was also decided that the mineralizing additives would be boron and fluorine compounds in the form of H_3_BO_3_ and NH_4_F, (POCH, Poland S.A., Gliwice, Poland) in such quantities that, calculated as pure boron and fluorine, their content in the dry set should be 1% and 3% by weight, respectively. The samples were soaked in the appropriate amount of distilled water, in which the proper amounts of additives were dissolved. After drying the moist samples, along with the additives, they were subjected to preliminary decarbonization at 100 °C for 30 min [17,18].

The following heating temperatures [°C] were selected: 1100, 1200, 1250, 1300, 1400, 1500, and correspondingly chosen synthesis times: 15, 30, 45, 60, and 90 min. Not all samples were subjected to the same synthesis temperatures, as shown in Table 1. The prepared samples were then subjected to the appropriate thermal treatment process at temperatures according to Table 2.

The sintered samples were subjected to the grinding process in an agate mill to ensure the appropriate grain size of the preparations. Each of the samples weighing 6 g, was moistened with water at a water-to-cement ratio W/C = 0.4 and at a constant room temperature of 20 °C (±1 °C), then placed in closed plastic containers. For the prepared preparations, the hydration process occurred at intervals: 1 day, 3 days, 7 days, 14 days, and 28 days. After each established period, the reacted samples were ground using an agate mortar, and subsequently, they could undergo comprehensive X-ray analysis [2,18].

### 2.2. XRD Analysis

The primary research technique utilized in this study was qualitative and quantitative analysis performed using the XRD (X-ray diffraction) method. The process of complete analysis was divided into several stages [19]:

- Qualitative examination of the phase compositions of pure sintered materials and pastes (without mineralizing additives) of the preparations;
- Qualitative examination of the phase compositions of samples doped with boron and fluorine;
- Determination and adoption of appropriate parameters for quantitative XRD methods;
- Quantitative X-ray analysis of both types of preparations.

The samples obtained after thermal treatment and hydration, when using the powder X-ray diffraction method, were additionally ground in an agate mortar to achieve the proper degree of comminution. All tested preparations must have the same degree of comminution.

Preparations made as above were subjected to qualitative and quantitative analysis of the phase composition using XRD methods. X-ray analyses were performed on a “Philips” apparatus. Qualitative studies were conducted in the range of angles from 5 to 75° 2θ, while quantitative studies of preparations were carried out in the ranges of occurrence of selected reflexes. A Cu lamp was used with the following settings:

- lamp voltage: 45 kV;
- filament current: 25 mA.

Parameters for qualitative analysis:

- angular range: from 5 to 75° 2θ;
- step counting mode;
- step size of the diffractometer arm: 0.05° 2θ;
- counting time: 2 s;
- powder sample—rotating.

Parameters for quantitative analysis:

- step counting mode,
- step size of the diffractometer arm: 0.02° 2θ,
- counting time: 10 s,
- powder sample—rotating,
- minimum number of repetitions: ×3.

To interpret the experimental results, PANalytical software HighScore Plus (Version 2.1.0) was used. Qualitative and quantitative analysis (excluding the amorphous phase) was carried out to provide a more precise assessment of the phase composition, using predefined parameters. It was not necessary to use an internal standard. This method helped to avoid additional dilution of the analyzed samples, which contributed to increased precision in the identification of doped phases.

Wykorzystano konkretne pliki CIF do analizy, w tym: α—Al_2_O_3_—63648.cif; CaO—26,959.cif; 3CaO·Al_2_O_3_—1841.cif; 12CaO·7Al_2_O_3_—6287.cif; CaO·Al_2_O_3_—260.cif; CaO·2Al_2_O_3_—14,270.cif i Ca_3_(BO_3_)_2_—23,664.cif [20]. Reference phase structural data available in the ICSD (International Crystal Structure Database) databases were used to determine the content of individual phases in the samples. Specific CIF files for analysis were used, including: α—Al_2_O_3_—63,648.cif; CaO—26,959.cif; 3CaO·Al_2_O_3_—1841.cif; 12CaO·7Al_2_O_3_—6287.cif; CaO·Al_2_O_3_—260.cif; CaO·2Al_2_O_3_—14,270.cif and Ca_3_(BO_3_)_2_—23,664.cif [20].

The values obtained as the quantitative analysis were rounded to the nearest 0.5% according to the declared level of accuracy of the quantitative measurements, thus ensuring the reliability of the analysis.

Based on the information available in the help section of the programme, the reliability of the quantitative results is assessed by the GOF (Goodness of Fit) coefficient, which should range from 1 to a maximum of 5. The lower the GOF coefficient value, the more reliable the test results are. In the case of our study, GOF fit coefficients ranged from 1.5 to a maximum of 2.5, indicating a high reliability of the results obtained. For quantitative analysis, the computer program “Analyze”, Rayflex Version 1.0, was used [20,21].

### 2.3. Quantitative and Statistical Analysis

The research results, including the percentage shares of calcium carbonate in the tested samples obtained using quantitative X-ray diffraction (XRD) analysis, were subjected to further quantitative analysis using the external standard method. This method relied on comparing the surface area of the analytical peak with the surface area of the standard peak [22].

In the case of paste analyses, it was assumed that the degree of hydration “α” of the analyzed calcium aluminate phases (CA_2_) was determined as the ratio of the surface area of the selected peaks for specified values of “d” in [Å] for hydrated samples to the surface area of peaks for identical values of “d” in [Å] for samples not subjected to the hydration process (standard 100%). A preparation with a calcium carbonate content of 100% was used as standard. The surface areas of the selected peaks were determined using the computer program “Analyze” [20]. The calculation of the content of the unreacted phase was performed based on the formula:

*X* = (100%⋅*Y*/*Z*)%, (3)

where:

*Y*—the surface area of the respective peak for the calculated hydrate (1 day, 3 days, 7 days, 14 and 28 days),
*Z*—the surface area of the respective peaks for the reference cement, not subjected to hydration.

To determine the degree of hydration “α” (the quantity of reacted phase), the differences between the content of a given phase in the reference (initial) sample (assuming 100% content of CA_2_, regardless of the actual content of this phase in the compound) and the content of the given phase in the cement subjected to hydration process were calculated:

*α* = 100% − *X*, (4)

The results of the quantitative analysis were statistically processed using the computer program “Statistica v 5.0 PL” and presented in tabular form. Additionally, these results were depicted in illustrating the relationship between the degree of synthesis at assumed temperatures, the degree of reaction “α”, and the duration of the hydration process. It was assumed, that when presenting this relationship as graphs, the degree of hydration “α” represents the average value of statistical calculations. According to the literature data [23], the error of the quantitative determination method using X-ray techniques was determined to be ±2%. The mean values, medians, standard deviations, and standard errors were calculated with a confidence level of 95%. The number of measurements in the quantitative analysis is not proportional to the amount of obtained results because samples with 100% CA_2_ content (i.e., full synthesis of calcium carbonate) were not included in the analysis.

### 2.4. Thermogravimetric Analysis

The thermogravimetric tests carried out aimed to monitor the temperature-dependent changes in the mass of the sample to accurately quantify the hydration products of the calcium aluminate phases. The mass loss analysis was carried out using a NEXTA STA200 thermogravimetric analyzer (Hitachinaka, Ibaraki, Japan). Each 3 g sample was placed in a platinum crucible and then thermally treated in an oven connected to a balance. The heating process took place in stages, where the sample was gradually heated at a constant oven temperature rise rate of 5 °C/min until the set temperature of 1000 °C was reached.

During the experiment, the temperature was measured using a thermocouple placed near the sample vessel to ensure the accuracy of the measurements. The entire measurement was carried out in a gaseous atmosphere, where air was used as the carrier gas. The use of such an environment was important to obtain reliable data on sample mass changes. The present study was crucial for the quantitative analysis of the hydration products of calcium aluminate phases, an important aspect in many scientific and industrial fields. Accurate thermogravimetric measurements provided valuable information that can be used for further research and optimisation of processes related to ceramic materials and other fields where control of thermal reactions is crucial.

### 2.5. Morphology Studies

The morphology of the samples was examined using an ultra-high-resolution scanning electron microscope (FEI, Nova NanoSEM 200, Philips, Eindhoven, The Netherlands), equipped with a thermal field emission electron gun (FEG-Schottky emitter, Philips, Eindhoven, The Netherlands), operating at an accelerating voltage of 18 kV. The chemical composition of the tested samples was identified using an X-ray energy dispersion analyzer (EDS) from EDAX Genesis XM, connected to the scanning electron microscope.

## 3. Results and Discussion

### 3.1. XRD Analysis Results

**Figure 1:**
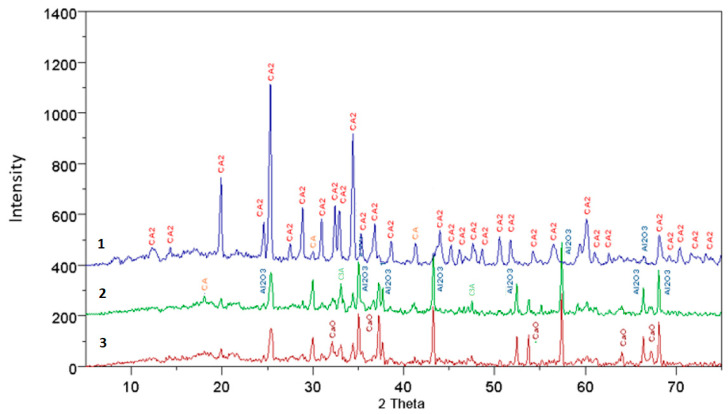
The diffraction pattern for a synthesis time of 15 min at temperatures of 3—1200 °C, 2—1300 °C, and 1—1500 °C, for samples without additives.

**Figure 2:**
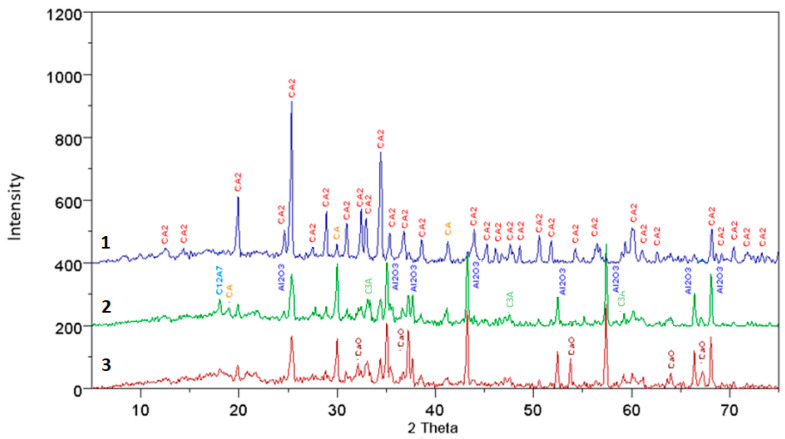
The diffraction pattern for a synthesis time of 90 min at temperatures of 3—1200 °C, 2—1300 °C, and 1—1500 °C, for samples without additives.

**Figure 3:**
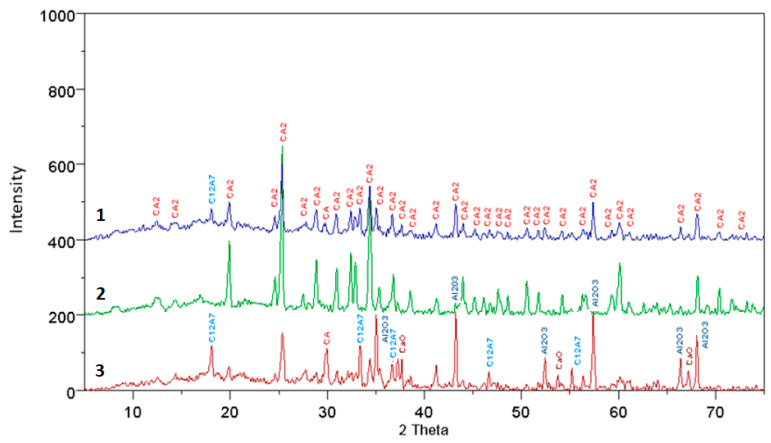
The diffraction pattern for a synthesis time of 15 min at temperatures of 1—1100 °C with 3% fluorine additives, 2—1200 °C with fluorine additives, and 3—1100 °C with 1% fluorine additives.

**Figure 4:**
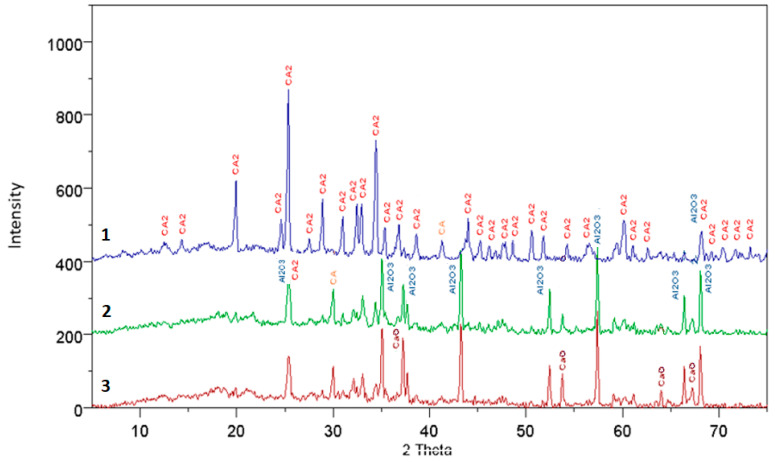
The diffraction pattern for synthesis times of 15, 45, and 90 min at temperatures of 3—1200 °C, 2—1250 °C, and 1—1300 °C, with a 1% boron additive.

The results of the XRD analysis, presented in the form of diffraction patterns, were interpreted and described to interpret the present phases. The diffraction patterns are shown in Figure 1, Figure 2, Figure 3 and Figure 4, where individual peaks were assigned to corresponding phases and described briefly along with their d-spacing values [Ǻ]. The analysis of the obtained diffraction patterns also allowed for a partial assessment of the content of other mineral phases present in the samples, besides calcium aluminate, such as CaO, C_3_A, C_12_A_7_, CA, CA_2_, and Al_2_O_3_—PDF: 43-1001; 9-413; 34-440; 23-1037 and 46-1212.

The results of this analysis are presented and compiled in Table 3, Table 4, Table 5, Table 6, Table 7, Table 8, Table 9, Table 10, Table 11, Table 12 and Table 13. Based on a commonly used, approximate method, a comparison of the intensity of reflections and the area of individual peaks (without using standards) was carried out to estimate semi-quantitatively the content of each phase.

The results of the qualitative analysis of fully reacted samples, compared to pure samples and those doped with mineralizers, show that they consist solely of the CaO·2Al_2_O_3_ phase. In the case of incomplete synthesis, in samples subjected to thermal treatment, besides the main phase, other compounds were also present, such as CaO, C_3_A, C_12_A_7_, CA, CA_2_, αAl_2_O_3_, and Ca_3_(BO_3_)_2_.

**Figure 5:**
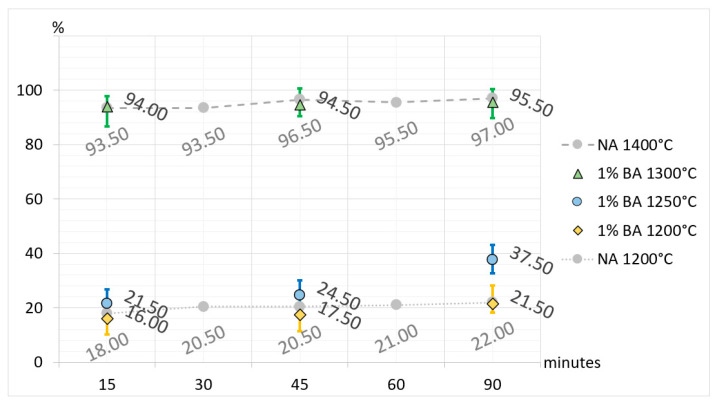
Comparison of the amount of CA_2_ in the sets without additives (NA) and sets with 1% boron compound additive (BA) with regard to the synthesis time and temperature.

**Figure 6:**
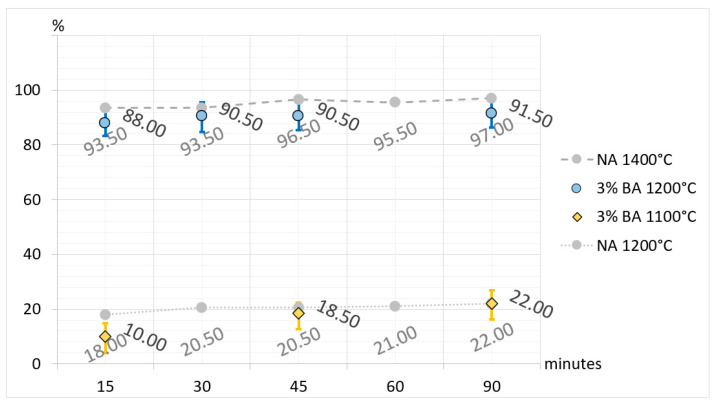
Comparison of the amount of CA_2_ in the sets without additives (NA) and sets with 3% boron compound additive (BA) with regard to the synthesis time and temperature.

**Figure 7:**
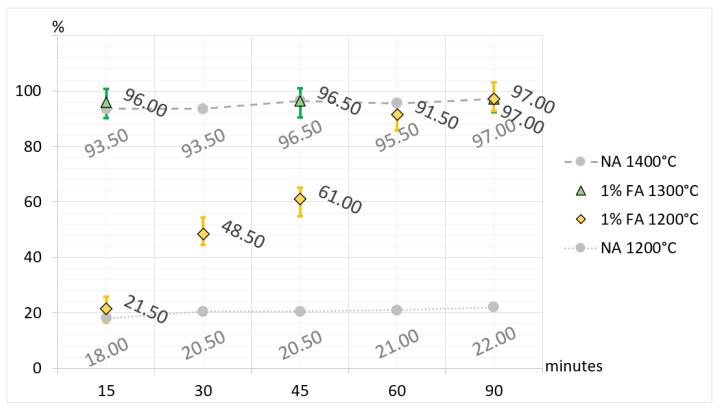
Comparison of the amount of CA_2_ in the sets without additives (NA) and sets with 1% fluorine compound additive (FA) with regard to synthesis time and temperature.

**Figure 8:**
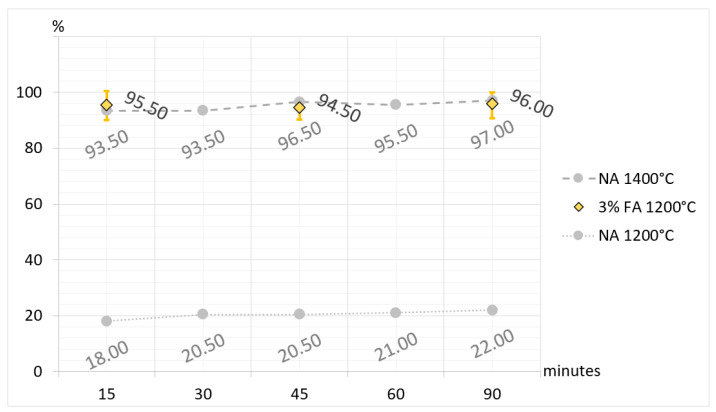
Comparison of the amount of CA_2_ in the sets without additives (NA) and sets with 3% fluorine compound additive (FA) with regard to synthesis time and temperature.

Additionally, to broaden interpretational possibilities and facilitate a direct comparison of CA_2_ synthesis, selected results are presented in Figure 5, Figure 6, Figure 7 and Figure 8. The charts are organized according to the convention, where the horizontal axes represent synthesis times and the vertical axes represent the percentage of synthesized CA_2_ in samples. In the charts, the synthesis of CA_2_ without additives in temperatures of 1200 °C and 1400 °C provides a level of reference (compare with Table 3 and Table 5) for comparison with synthesis with boron compound or fluorine compound additives. In the case of synthesis with boron compound or fluorine compound additives, error bars have been added to the graphs. To make the bars visible, their values are magnified by five times.

For samples not doped with mineralizers, at synthesis temperatures of 1200 °C and 1300 °C, all mentioned phases are present in the analyzed specimens, except for calcium borate. However, at higher temperatures the phases CaO, C_3_A, and C_12_A_7_ gradually disappear, followed by CA and Al_2_O_3_ phases. It is worth noting that in the case of samples doped with boron additives, only the specimen synthesized at 1100 °C with a 3% boron additive contained, in addition to other minerals, the phase calcium borate Ca_3_(BO_3_)_2_, detectable by X-ray diffraction. In specimens doped with 3% boron and subjected to higher temperatures, the presence of this compound was not observed. Specimens doped with 1% boron synthesized at 1200 °C and 1300 °C consist of phases CA, CA_2_, and αAl_2_O_3_. Similarly, in the case of synthesis with a 3% boron addition, the same phases are present, but at lower thermal treatment temperatures, i.e., 1100 °C and 1200 °C.

An interesting observation is that in the case of specimens doped with a fluorine compound, throughout the entire range of applied temperatures and thermal treatment times, no presence of fluorine compounds with CaO and Al_2_O_3_ was detected. Only during the synthesis of specimens at 1200 °C with a 1% fluorine addition, the examined samples consisted of CaO, C_12_A_7_, CA, CA_2_, and αAl_2_O_3_. In other cases at temperatures of 1200 °C and 1300 °C, complete synthesis of calcium aluminate CA_2_ occurred.

The analysis of the results presented in the tabular form (Table 9, Table 10, Table 11 and Table 12) for specimens without additives and with mineralizing additives indicates that in most cases, complete reaction of the samples was achieved after approximately 60 min of thermal treatment.

The presented and discussed results of the analysis of Figure 5, Figure 6 and Figure 7 allow us to conclude that doping the primary raw material set with boron and fluorine compounds reduces and accelerates the synthesis temperature of calcium aluminate CA_2_. Taking the temperature of 1500 °C as a reference point for the full synthesis temperature of CA_2_ for the set without mineralizers, it can be demonstrated that the addition of fluorine has a stronger effect on reducing the temperature and speeding up the synthesis. For a 3% fluorine admixture, the temperature of full CA_2_ synthesis was 1100 °C, and for a 1% addition, it was 1200 °C. Doping with boron in an amount of 3% resulted in reducing the synthesis temperature of calcium aluminate to 1200 °C, and for a 1% addition, it was 1300 °C. The discussed results apply to cases of thermal treatment lasting 60 and 90 min. It is also important to remember that the achieved results of temperature reduction in synthesis apply only to the case of the raw material set prepared according to the description in this study.

The obtained results of the statistical analysis indicate that for all considered cases, determining the percentage content of calcium aluminate using XRD techniques is associated with fluctuations in the standard deviation values ranging from 0.75 to 4.30, while the standard error values range from 0.3 to 2.4.

### 3.2. Research on the Unit Cell

Concurrently with the qualitative–quantitative XRD studies, an initial attempt was made to determine the parameters of the unit cell of the obtained samples to demonstrate potential differences in the structure and dimensions of the CA_2_ phase unit cell, particularly in the case of doping the raw material set with mineralizers. The parameters of the unit cells were determined based on two specialized computer programs: “Powder X” and “Unitcell”. Each of these programs required a separate file from the obtained diffractometric data. The results of the preliminary studies of the unit cells of the “pure” calcium aluminate phase and the phase doped with mineralizing additives are presented in table below.

The analysis of the results presented in Table 14 leads to the conclusion that the determined parameters of the unit cells obtained in the experiment for the CA_2_ phases do not deviate from the parameters of the unit cells of phases with calcium aluminate as reported in the cited literature [14]. This suggests that both the quality and quantity of the introduced mineralizing additives do not cause changes in the parameters of the unit cells of the obtained samples.

### 3.3. Hydration Studies

The qualitative X-ray analysis showed that the samples obtained through thermal treatment of both “pure” and mineralized sets consist solely of the phase CaO·2Al_2_O_3_ (marked with X in Table 15). Since no other phases were found in the examined samples besides calcium aluminate, it was assumed that the synthesis process of this phase was complete. The temperature of full synthesis of calcium aluminate (CA_2_) for the “pure” set without mineralizers was determined to be 1500 °C with a thermal treatment duration of 60 min (see Table 15). A detailed qualitative XRD analysis of the results indicates that no other phases are present in the examined samples besides the calcium aluminate phase. This observation suggests that in the case of “pure” samples and those mineralized with additives, phases resulting from reactions between calcium oxide and boron compounds or calcium oxide and fluorine compounds do not form, nor do phases formed from aluminum oxide compounds with boron compounds or aluminum oxide compounds with fluorine compounds [13].

The above discussion should also be applied to the formation of ternary compounds consisting of oxides B_2_O_3_, Al_2_O_3_, and CaO, and also compounds resulting from the reaction of a fluoride compound with aluminum oxide and calcium oxide [12,13,25]. Doping the raw material set with a 1% (by weight) fluoride compound resulted in the completed synthesis of the CA_2_ phase at 1300 °C, while in the raw material set with a 1% boron compound, complete synthesis of calcium aluminate occurred at 1200 °C. The completed reaction of samples for the “pure” CA_2_ phase and for the CA_2_ phase with fluoride occurred after 60 min of thermal treatment at the mentioned temperatures, whereas for the CA_2_ phase with boron, it was achieved after 90 min. Preliminary studies of the unit cell parameters of the “pure” CA_2_ compound and phases doped with selected mineralizers demonstrate full compliance with data in the scientific literature [13,26]. Interestingly, the addition of boron or fluorine compounds in an amount not exceeding 1% does not induce changes in the unit cell parameters corresponding to the dimensions of the “pure” CA_2_ phase. These findings are supported by the analysis of the results presented in Table 14. The unit cell parameters of the “pure” CA_2_ phase align with those of phases doped with mineralizers, and the differences in dimensions between literature data and those obtained in this study range from 0.02 to 0.04%, falling within the measurement error range. Examining the hydration process of the obtained calcium aluminate preparations, as shown in Figure 8 and Table 16, Table 17, Table 18 and Table 19, it can be observed that for the “pure” CA_2_ phase, after 1 day of reaction with water, the degree of reaction is approximately 15%. By the third day of hardening, this degree reaches about 35%, gradually increasing over time and reaching a maximum value of about 62% after 28 days of the hydration process [27]. For the CA_2_ phase with a 1% boron addition, initially, slight progress in the hydration process is observed, resulting in a degree of reaction of about 10% after the first day. In the time interval between the first and third day of the hydration process, the increase in the hydration degree “α” for this phase is minimal, amounting to only about 5%. After 3 hydration days, there was a significant acceleration of this process, with the determined value of “α” after 7 days being about 47% [28,29]. Between 7 and 28 days of reaction of this phase with water, there is a gradual slowing down of the pace of this process, reaching only a 50% degree of reaction after 28 days. Observing the hydration process of the CA_2_ phase doped with a 1% fluoride addition, it can be noted that after one day of reaction with water, only a 7% degree of reaction is achieved, and then in the interval between the first and seventh day of the hydration process, the level reached is 53% [27]. Between the 7th and 28th day of the water reaction process, a stabilization of the pace of this process is observed, which reaches approximately 70% reaction completion at the final stage, after 28 days [16,30,31].

**Figure 9:**
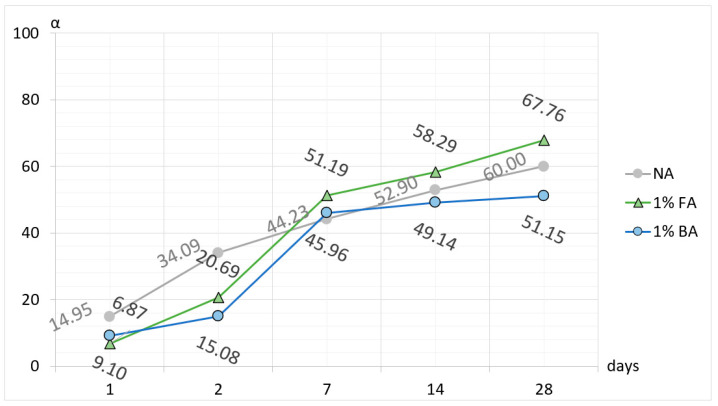
The time dependence of the hydration level (α in %) of CA_2_ without additives (NA) as well as CA_2_ with boron (BA) and fluorine (FA) compounds.

Analyzing the relationship between the hydration time of the samples and the degree of reaction “α” of the CA_2_ phases leads to the conclusion that for the “pure” CA_2_ preparation without mineralizers, the hydration process is faster in the period from 1 to 3 days after water mixing compared to preparations doped with boron and fluoride compounds (Figure 9). For phases doped with fluoride and boron compounds, a faster increase in the hydration process rate is observed between 3 and 7 days. After 7 days of hydration, both the “pure” preparation and the preparations doped with mineralizers have similar hydration degrees “α”, ranging from 45% to about 51%. The addition of fluoride compounds induces a more intense reaction with water for the CA_2_ phase doped with fluoride than for the “pure” phase. Conversely, in the case of doping the CA_2_ phase with boron compounds, the reaction process with water is slower compared to the “pure” calcium aluminate phase. Regarding the dynamics of the hydration process of the “pure” CA_2_ phase, it can be stated that doping this phase with fluoride compounds accelerates hydration, which consequently may lead to higher mechanical properties of the cement paste. Conversely, doping the “pure” CA_2_ phase with boron compounds may lead to the opposite effect. Slower hydration processes of this phase may result in slower hydration processes overall. In summary, it can be concluded that doping the basic raw material set with boron and fluoride compounds reduces and accelerates the synthesis temperature of calcium aluminate. If we take the temperature of 1500 °C as the reference point for the full synthesis temperature of the CA_2_ phase for the set without mineralizers, we can demonstrate that boron addition has a stronger effect on reducing the synthesis temperature. Doping with 1% boron compound resulted in lowering the synthesis temperature of calcium aluminate to 1200 °C.

The quantitative analysis of the hydrated samples was complemented by thermogravimetric analysis of the sets containing calcium bicarbonate, both pure and doped with ions modifying the synthesis temperature. A detailed analysis is included in Table 20. The results of the quantitative analysis, carried out using the X-ray method, in comparison with the thermogravimetric analysis, show a reasonably high agreement. Higher percentages of mass loss are recorded for the TG method. The information obtained indicates that, in addition to the crystalline phases determined by powder methods, there are amorphous or poorly crystallized phases, which can certainly include aluminum hydroxides.

Samples of calcium aluminates were subjected to hydration processes in order to demonstrate the influence of the addition of modifying ions on the synthesis temperature of the preparations as well as on the morphology of the hydration products. Microstructure analysis was carried out after 1, 3, 7 and 14 days of the hydration process. For the pure phase without modifying additives, the analysis was carried out after 1 and 14 days of the hydration process. In the case of samples doped with 1% boron compound and 1% fluorine compound, the analysis of the microstructure image of the sample was carried out after 3 and 7 days according to the order used previously. In addition to the standard images showing the sample microstructure, X-ray surface elemental distribution (samples 1 and 3) and quantitative elemental analysis of the micro-areas (samples 2 and 4) were performed. Microstructure studies were carried out according to the quantitative and qualitative analysis of the hydrated samples, as described in Section 3.3.

**Figure 10:**
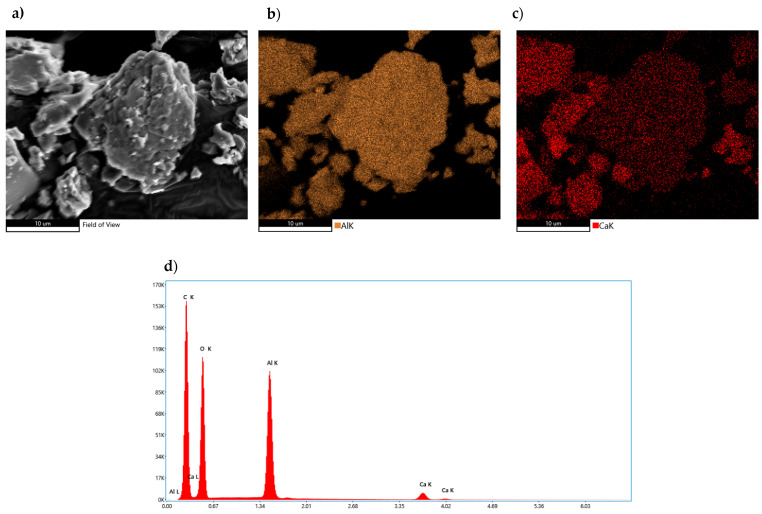
Morphology of CA_2_ sample with analysis of the distribution of elements on its surface and average quantitative analysis of the studied micro-area. (**a**) SEM photo of the tested sample, (**b**) Al concentration, (**c**) Ca concentration, (**d**) summary EDS analysis of elements in the examined area.

Figure 10 shows the morphology of the non-hydrated CA_2_ phase (panels a–c). Quantitative sample analysis based on the EDS method in the mapped areas showed visible changes in the elemental concentration of calcium (highlighted in red in panel c), particularly at the left edge of the image.

These observations correlate with the image showing the location of the aluminum element, clearly lowering its concentration in areas with higher calcium elemental content. These results are consistent with the qualitative analysis performed by XRD (panel d), as the sample, in addition to the predominant CA_2_ phase content, also contains aluminum-poor and calcium-rich phases, such as C_3_A and C_12_A_7_. Quantitative analysis of the average concentration values of the abovementioned elements from the entire sample surface confirms the domination of the aluminum-rich phase.

**Figure 11:**
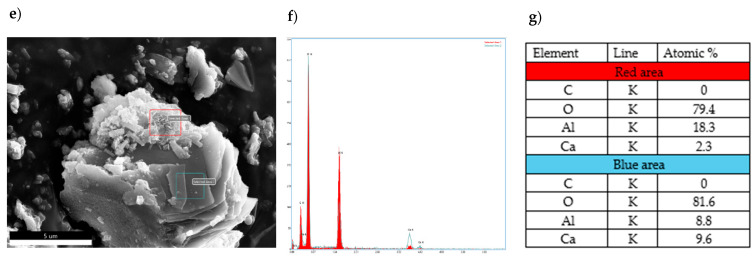
Morphology of CA_2_ sample subjected to hydration process for 1 day with analysis of the distribution of elements on its surface and average quantitative analysis of the studied micro-area. (**e**) SEM photo of the tested sample, (**f**) EDS analysis of elements in the examined (red and blue) areas, (**g**) quantitative elemental composition of the areas examined in the SEM image.

Figure 11 shows the morphology of CA_2_ without the addition of modifying compounds (panel e) after 1 day of the hydration process. An analysis of the concentration of elements present in the examined micro-areas on the samples’ surface (panel f) and an average elemental composition analysis were carried out (panel g). The microphotograph of the sample shows a grain, on the surface of which two areas with a microstructure showing fundamental differences can be identified. The area in the red box is characterized by the presence of overlapping lamellar conglomerate plates growing from the grain surface (most likely products of hexagonal calcium aluminates), resembling crumpled films.

The blue-framed area represents a flat surface not assigned to any particular crystalline phase. The analysis of the micro-areas combined with the quantitative EDS plot showed a fundamental difference in the aluminum content between the examined areas, with 52% less aluminum present in the area marked with the blue frame compared to the area marked with the red frame. This fact may indicate that hydrated calcium aluminates with different Al/Ca atomic ratios are present in the sample in addition to the non-hydrated hydration products. The varying Al/Ca ratios may also be related to the fact that the phase composition of the sample before hydration was varied, with compounds such as C_3_A, C_12_A_7_, and CA present in addition to the predominant CA_2_ phase content.

**Figure 12:**
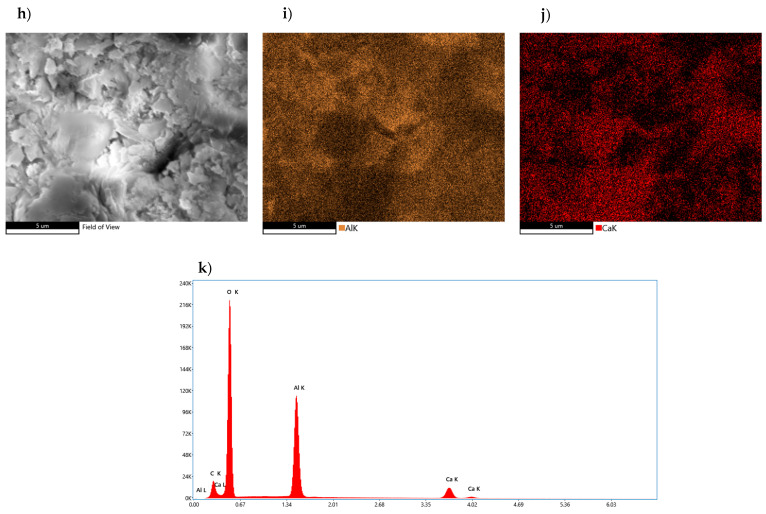
Morphology of CA_2_ sample doped with 1% of fluoride compound subjected to hydration process for 7 days with analysis of the distribution of elements on its surface and average quantitative analysis of the studied micro-area. (**h**) SEM photo of the tested sample, (**i**) Al concentration, (**j**) Ca concentration, (**k**) summary EDS analysis of elements in the examined area.

Further microscopic investigations focused on the analysis of a sample containing 1% fluoride compound, in which the hydration process was stopped on its seventh day (Figure 12h–j). Elemental concentrations on the samples’ surface were mapped and an average elemental composition analysis was presented (panel k). Photographs of the surface showed the presence of a very compact, non-porous microstructure with varying crystal shapes. Cascading lamellar crystals exceeding the size of 1 μm are noticeable on the sample, with much smaller clusters of lamellar crystals in between, whose agglomerations resemble the image of a bent film. In the case of the sample in question, there was a high homogeneity of areas showing concentrations of calcium and aluminum. In the case of the aluminum element, there are local areas where its quantity is higher. XRD analysis confirmed the above findings, showing that the composition is dominated by the CA_2_ phase, with CA and Al_2_O_3_ also present. The resulting mean micro-area X-ray dispersion analysis showed a significantly higher ratio of aluminum to calcium, thus confirming the presence of aluminum-rich compounds. The analyzed sample fragment did not show the presence of fluorine compounds.

**Figure 13:**
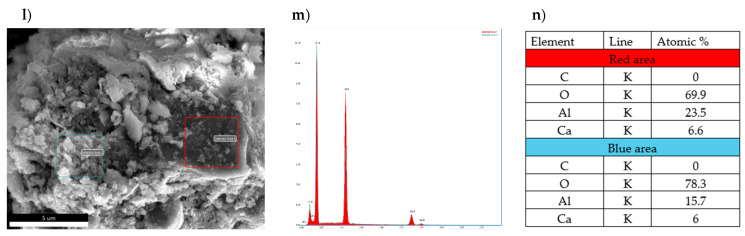
Morphology of CA_2_ sample doped with 1% of boron compound subjected to hydration process for 3 days with analysis of the distribution of elements on its surface and average quantitative analysis of the studied micro-area. (**l**) SEM photo of the tested sample, (**m**) EDS analysis of elements in the examined (red and blue) areas, (**n**) quantitative elemental composition of the areas examined in the SEM image.

In the photographs of the sample CA_2_ doped with 1% of boron compound subjected to hydration process for 3 days (Figure 13), one can observe the occurrence of a varied microstructure in which small areas are distinguished, forming visible clusters of crystallites with diameters much smaller than 1 micrometer. In some places, large lamellar sheets located perpendicular to the flat surface of the grain can be observed. The conglomerates of small-sized crystallites resemble a spherical shape giving rise to the assumption that these are regular hydrated calcium aluminates formed by local conversion of hexagonal crystals. Spot atomic analysis of the chemical composition of selected areas in the sample was carried out. It can be concluded that for both the red and blue areas there are no significant differences in elemental composition although a higher aluminum content was found in the red rectangle.

The same relationship is apparent in the EDS spectra. Due to the chemical composition of the analyzed sample, it is reasonable to find higher aluminum contents in relation to calcium. X-ray dispersion analysis shows that there are no boron-derived elements in the studied area.

**Figure 14:**
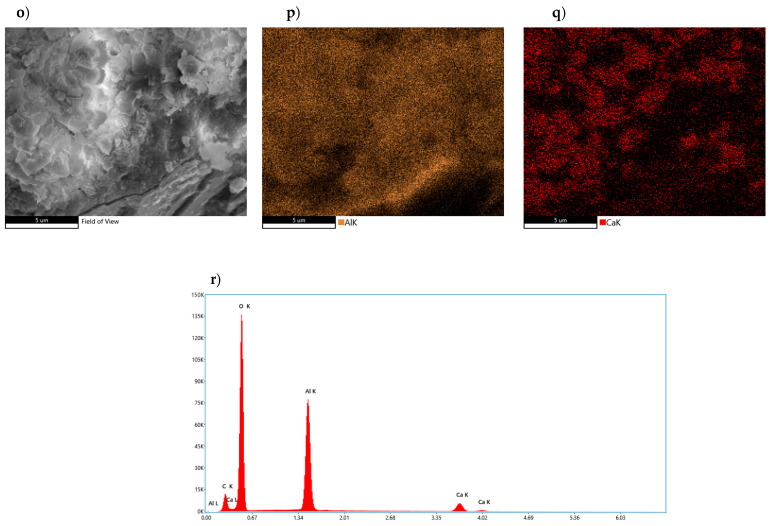
Morphology of CA_2_ sample subjected to hydration process for 14 days with analysis of the distribution of elements on its surface and average quantitative analysis of the studied micro-area. (**o**) SEM photo of the tested sample, (**p**) Al concentration, (**q**) Ca concentration, (**r**) summary EDS analysis of elements in the examined area.

Analysis of the microstructure of the CA_2_ sample after 14 days of the hydration process (panel o) revealed the presence of a rather compact microstructure (Figure 14). In it, hexagonal layers of lamellae and clusters of cubic phases can be observed, which are accumulated in the compact surface of the test sample. In order to show the variation and the concentration of atoms, the entire study area was mapped. The “p” panel shows the concentration of aluminum atoms, while the “q‘‘ panel presents the arrangement of calcium atoms on the sample surface. Analysis of the morphology of the sample showed heterogeneity in the distribution of atoms in the different areas. In the image showing the areas of calcium atoms, areas of higher concentration of calcium atoms can be clearly seen, while this relationship is not so apparent for aluminum atoms. This relationship suggests the presence of different hydration products, which vary in calcium ion content. The average content of the individual atoms in the sample clearly indicates the dominance of aluminum atoms over calcium atoms.

The discussed research results pertain to cases of thermal treatment lasting 60 and 90 min. Analyzing the hydration process and evaluating the potential consequences caused by introducing mineralizing additives, it can be inferred that using fluoride compounds is a more favorable option than doping CA_2_ with boron compounds. Preferring the use of a fluorine-based mineralizer may lead to an increase in the mechanical properties typically achieved by the hardening paste.

Considering the above, it can be assumed that the research objective of accurately selecting the type and amount of additives has resulted in a significant reduction in the synthesis temperature of CA_2_ phases, as well as provided various insights into the speed and dynamics of the hydration process between water and the “pure” and boron- and fluoride-doped calcium aluminate phases (CaO·2Al_2_O_3_).

## 4. Conclusions

Based on the conducted research and analysis of the obtained results, the following scientific conclusions can be drawn:

- The full synthesis temperature of calcium aluminate (CA_2_) for the raw material set without mineralizing additives was 1500 °C.
- Adding a fluoride compound to the raw material set, with a 3% admixture, resulted in full synthesis of CA_2_ already at a temperature of 1100 °C, while with a 1% addition, this temperature was 1200 °C.
- Adding a boron compound to the raw material set, with a 3% admixture, led to full synthesis of CA_2_ already at a temperature of 1200 °C, while with a 1% addition, this temperature was 1300 °C.
- Complete reaction of the samples for all examined cases was achieved after approximately 60 min of thermal treatment at the aforementioned temperatures.
- Adding a fluoride compound to the raw material set, over the entire range of temperatures and thermal treatment times, did not lead to the formation of fluoride compounds with CaO and Al_2_O_3_. Only the preparation synthesized at 1100 °C with a 3% admixture contained, among other minerals, calcium borate phase (Ca_3_(BO_3_)_2_).
- The use of boron and fluoride compounds in amounts up to 1% as mineralizing additives in the synthesis processes of the CA_2_ phase does not cause changes in the unit cell parameters of the obtained calcium aluminate phases.
- The degree of hydration of calcium aluminate phases without mineralizing additives ranges from 15 to 60% of the degree of reaction between 1 and 28 days of the process.
- The degree of hydration of calcium aluminate with a 1% fluoride addition ranges from 7 to 70% between 1 and 28 days of the process.
- The degree of hydration of calcium aluminate phases doped with boron compounds at 1% ranges from 9 to 51% between 1 and 28 days of the hydration process.
- Adding fluoride compounds to the CA_2_ phase accelerates the hydration process, which consequently may lead to better mechanical properties of the paste, while the addition of boron compounds results in a slowing down of the reaction process with water.

Limitations of the research are as follows:

- Although SEM studies were conducted, along with a detailed analysis of element mapping it is challenging to determine the precise quantities of fluorine or boron compounds that alter the elementary cell of calcium aluminate and their impact on altering the dynamics of the hydration process, as both compounds vaporize at temperatures around 800 °C.
- In the current state of the research it is impossible to determine the exact correlation between the temperature reduction during synthesis depending on the addition of temperature-lowering ions in calcium aluminates. That is due to the uncontrolled evaporation process of boron and fluorine compounds.
- As both, boron and fluoride compounds undergo evaporation processes, at this stage it is not possible to specifically indicate the percentage contribution or determine the mechanism of their reaction regarding the reduction of eutectic points in the studied systems.
- The authors intend to address the above issues in future studies.

The presented conclusions confirm the influence of fluoride and boron compound additives on the synthesis and hydration process of calcium aluminate. They also suggest further research to better understand and utilize these properties in practice. Further studies should focus on determining the levels of mineralizing additives where potential changes in the unit cell parameters of the forming phases could be observed. Additionally, efforts should be directed towards determining other physicochemical properties of hydrated calcium aluminate doped with fluoride and boron ions.

## Figures and Tables

**Table 1:** Physicochemical properties of the phases occurring in the CaO-Al_2_O_3_ system.

Phase	Physicochemical Properties
C_3_A	Melts incongruently at 1639 °C (5) into CaO and liquid phase with a composition of 57.2% CaO, 42.8% Al_2_O_3_, and forms an eutectic with CA at 1360 °C (5) with a composition of 49.35% CaO and 50.65% of Al_2_O_3_.
CA	Melts incongruently at 1602 °C (5) into CA_2_ and liquid phase with a composition of 36% CaO and 64% Al_2_O_3_. Forms an eutectic with C_3_A.
CA_2_	Melts incongruently at 1762 °C (±5) into CA_6_ and liquid phase consisted of 22% of CaO and 78% of Al_2_O.
CA_6_	Melts incongruently at 1830 °C into a corund and liquid phase consisted of 16% of CaO and 84% of Al_2_O_3_.
C_12_A_7_	In dry air melts incongruently at. 1374 °C into CA and liquid phase. In the air of normal humidity incongruently at 1392 °C.

**Table 2:** The selected synthesis temperatures for samples containing boron and fluoride compounds.

Sample ^1^	Synthesis Temperature					
	1100 [°C]	1200 [°C]	1250 [°C]	1300 [°C]	1400 [°C]	1500 [°C]
No additive	X	X		X	X	X
1% B		X	X	X		
3% B	X	X				
1% F		X		X		
3% F	X	X		X		

^1^ Percentages stand for weight content of pure boron (B) or fluoride (F) in the samples. X—Sample selected for synthesis in given temperature

**Table 3:** Identification of phases, synthesis temperature 1200 °C, no additives.

Synthesis Time [min]	CaO	C_3_A	C_12_A_7_	CA	CA_2_	α-Al_2_O_3_	GOF
15	8.0	6.0	3.5	4.5	18.0	50.0	1.21
30	6.5	5.5	3.0	15.5	20.5	49.0	1.55
45	6.5	5.5	3.0	15.5	20.5	49.0	1.62
60	6.5	5.5	3.5	15.5	21.0	48.0	1.75
90	5.0	5.5	4.5	17.5	22.0	45.5	1.72

**Table 4:** Identification of phases, synthesis temperature 1300 °C, no additives.

Synthesis Time [min]	CaO	C_3_A	C_12_A_7_	CA	CA_2_	α-Al_2_O_3_	GOF
15	4.5	5.0	7.0	17.0	18.5	48.0	1.55
30	3.5	4.5	7.0	18.0	18.0	49.0	1.61
45	3.0	4.5	6.0	20.5	18.5	47.5	1.57
60	2.5	4.0	6.0	21.5	19.0	47.0	1.50
90	1.5	3.5	6.0	24.5	21.5	43.0	1.45

**Table 5:** Identification of phases, synthesis temperature 1500 °C, no additives.

Synthesis Time [min]	CaO	C_3_A	C_12_A_7_	CA	CA_2_	α-Al_2_O_3_	GOF
15	0.0	1.5	1.0	2.0	93.5	2.0	2.00
30	0.0	1.0	1.0	2.5	93.5	2.0	2.08
45	0.0	0.5	0.5	1.5	96.5	1.0	2.31
60	0.0	1.0	0.5	2.0	95.5	1.0	2.07
90	0.0	0.5	0.0	1.5	97.0	1.0	1.96

**Table 6:** Identification of phases, synthesis temperature 1200 °C, 1% boron additive.

Synthesis Time [min]	CaO	C_3_A	C_12_A_7_	CA	CA_2_	α-Al_2_O_3_	GOF
15	7.0	4.5	2.5	15.5	16.0	54.5	1.70
45	4.5	5.0	4.0	20.5	17.5	48.5	1.47
90	3.0	4.5	5.0	21.0	21.5	45.0	1.44

**Table 7:** Identification of phases. synthesis temperature 1250 °C. 1% boron additive.

Synthesis Time [min]	CaO	C_3_A	C_12_A_7_	CA	CA_2_	α-Al_2_O_3_	GOF
15	5.0	5.0	4.0	17.5	21.5	47.0	1.50
45	3.5	3.0	4.5	24.0	24.5	40.5	1.60
90	1.0	2.5	4.5	26.5	37.5	28.0	1.58

**Table 8:** Identification of phases, synthesis temperature 1300 °C, 1% boron additive.

Synthesis Time [min]	CaO	C_3_A	C_12_A_7_	CA	CA_2_	α-Al_2_O_3_	GOF
15	0.0	2.0	1.0	1.5	94.0	1.5	1.61
45	0.0	1.0	1.0	2.5	94.5	1.0	1.57
90	0.0	0.5	1.0	2.0	95.5	1.0	2.08

**Table 9:** Identification of phases, synthesis temperature 1100 °C, 3% boron additive.

Synthesis Time [min]	CaO	C_3_A	C_12_A_7_	CA	CA_2_	α-Al_2_O_3_	Ca_3_(BO_3_)_2_	GOF
15	4.5	1.0	2.0	14.0	10.0	58.0	10.5	1.67
45	2.5	1.0	2.0	15.0	18.5	51.0	10.0	1.64
90	2.0	1.5	2.5	16.0	22.0	46.5	9.5	1.69

**Table 10:** Identification of phases, synthesis temperature 1200 °C, 3% boron additive.

Synthesis Time [min]	CaO	C_3_A	C_12_A_7_	CA	CA_2_	α-Al_2_O_3_	Ca_3_(BO_3_)_2_	GOF
15	0.0	0.0	0.0	1.5	88.0	10.0	0.5	1.48
30	0.0	1.0	1.0	1.5	90.5	5.5	0.5	1.51
45	0.5	0.0	1.0	2.5	90.5	5.0	0.5	1.78
90	0.5	0.0	1.5	2.5	91.5	3.0	1.0	1.56

**Table 11:** Identification of phases, synthesis temperature 1200 °C, 1% fluorine additive.

Synthesis Time [min]	CaO	C_3_A	C_12_A_7_	CA	CA_2_	α-Al_2_O_3_	GOF
15	3.0	1.0	15.0	16.0	21.5	43.5	1.67
30	2.5	0.5	5.0	12.5	48.5	31.0	1.48
45	1.5	1.0	8.5	7.5	61.0	20.5	1.77
60	1.0	1.0	3.0	2.5	91.5	1.0	2.11
90	0.5	0.5	1.0	0.5	97.0	0.5	2.06

**Table 12:** Identification of phases, synthesis temperature 1300 °C, 1% fluorine additive.

Synthesis Time [min]	CaO	C_3_A	C_12_A_7_	CA	CA_2_	α-Al_2_O_3_	GOF
15	0.0	0.0	1.0	1.5	96.0	1.5	2.02
45	0.0	0.0	0.0	2.5	96.5	1.0	1.77
90	0.0	0.0	0.0	2.0	97.0	1.0	2.08

**Table 13:** Identification of phases, synthesis temperature 1200 °C, 3% fluorine additive.

Synthesis Time [min]	CaO	C_3_A	C_12_A_7_	CA	CA_2_	α-Al_2_O_3_	GOF
15	0.0	2.0	0.5	0.5	95.5	1.5	1.61
45	0.0	1.0	1.0	2.5	94.5	1.0	1.57
90	0.0	1.0	1.0	1.5	96.0	0.5	1.92

**Table 14:** The unit cell parameters of CA_2_ according to the datasheets for the synthesized preparations calculated using the “Unitcell” computer program [24].

Phase	Unit Cell Parameters [Å]			
	a	b	c	β
According to the literature [6]	12.82	8.84	5.42	107.50
No additives	12.86	8.88	5.43	106.80
1% B	12.86	8.87	5.43	106.87
1% F	12.87	8.87	5.43	106.87

**Table 15:** The selected synthesis temperatures for samples subjected to hydration. Samples marked with X consist solely of the CaO·2Al_2_O_3_ phase. ^1^ No additive—phase CA_2_ synthesized without the addition of boron and fluorine, 1%B—phase CA_2_ synthesized with the addition of 1% boron, 1%F—phase CA_2_ synthesized with the addition of 1% fluorine.

Phase ^1^	Synthesis Temperature and Time			
	1300 [°C]		1500 [°C]	
	60 [min]	90 [min]	60 [min]	90 [min]
No additive	-	-	X	-
1% B	-	X	-	-
1% F	-	X	-	-

**Table 16:** Results of the statistical analysis for quantitative studies of the CA_2_ phase, no additives.

Hydration Time [Days]	Average	Median	Standard Deviation	Standard Error	Number of Samples
1	14.95	14.13	2.72	0.90	9
3	34.09	33.41	1.90	0.67	8
7	44.23	44.46	2.51	0.84	9
14	52.90	52.59	2.37	0.80	9
28	61.00	60.96	3.31	1.17	8

**Table 17:** Results of the statistical analysis for quantitative studies of the CA_2_ phase, 1%B additive.

Hydration Time [Days]	Average	Median	Standard Deviation	Standard Error	Number of Samples
1	9.10	8.94	1.65	0.58	8
3	15.08	13.35	2.86	1.08	7
7	45.96	45.35	1.63	0.54	9
14	49.14	49.27	1.58	0.53	9
28	51.15	50.88	1.96	0.74	7

**Table 18:** Results of the statistical analysis for quantitative studies of the CA_2_ phase, 1%F additive.

Hydration Time [Days]	Average	Median	Standard Deviation	Standard Error	Number of Samples
1	6.87	14.13	2.72	0.90	9
3	20.69	33.41	1.90	0.67	8
7	51.19	44.46	2.51	0.84	9
14	58.29	52.59	2.37	0.80	9
28	67.76	60.96	3.31	1.17	8

**Table 19:** Results of the hydration analysis.

Phase	Degree of Hydration α [%]				
	1	2	7	14	28
No additives	14.95	34.09	44.23	52.90	60.00
1%F	6.87	20.69	51.19	58.29	67.76
1% B	9.10	15.08	45.96	49.14	51.15

**Table 20:** Results of thermogravimetric analysis of calcium bicarbonate samples sintered at 1000 °C [wt%].

Hydration Time [Days]	CA_2_ Phase, No Additives	CA_2_ Phase, 1%B Additive	CA_2_ Phase, 1%F Additive
1	15.24	9.45	7.08
3	35.59	15.83	21.72
7	47.90	46.58	55.79
14	54.25	50.19	62.62
28	64.44	52.42	71.86

## Data Availability

All measurement data are included in this publication. For enquiries, please contact michal.pyzalski@agh.edu.pl.
